# National perspectives of barriers by insurance and pharmacy benefit managers in pediatric inflammatory bowel disease

**DOI:** 10.1002/jpr3.70004

**Published:** 2025-02-10

**Authors:** Brad D. Constant, Jeremy Adler, Benjamin D. Gold, Jennifer Dotson, Jenifer R. Lightdale, Frank Scott, Shehzad Saeed, Sandra Kim, Jonathan Moses, Edwin F. de Zoeten, Lucia Mirea, Andrew Ritchey, Brad Pasternak

**Affiliations:** ^1^ Division of Pediatrics and Section of Pediatric Gastroenterology, Hepatology and Nutrition, Digestive Health Institute, Children's Hospital Colorado University of Colorado Anschutz School of Medicine, Medical Campus Aurora Colorado USA; ^2^ Department of Pediatrics, Division of Pediatric Gastroenterology, Susan B. Meister Child Health Evaluation and Research Center University of Michigan Ann Arbor Michigan USA; ^3^ GI Care for Kids, Children's Center for Digestive Healthcare, LLC Atlanta Georgia USA; ^4^ Pediatric Gastroenterology Arkansas Children's Hospital/University of Arkansas for Medical Sciences Little Rock Arkansas United States; ^5^ Division of Gastroenterology, Hepatology and Nutrition Boston Children's Hospital Boston Massachusetts USA; ^6^ Division of Gastroenterology and Hepatology University of Colorado Anschutz Medical Campus Aurora Colorado USA; ^7^ Department of Medical Affairs, Dayton Children's Hospital and Boonshoft School of Medicine Wright State University Dayton Ohio USA; ^8^ Division of Pediatric Gastroenterology Cleveland Clinic Foundation Cleveland Ohio USA; ^9^ Division of Pediatric Gastroenterology Stanford University Palo Alto California USA; ^10^ Division of Pediatrics and Section of Pediatric Gastroenterology, Hepatology and Nutrition, Digestive Health Institute, Children's Hospital Colorado University of Colorado Anschutz School of Medicine Aurora Colorado USA; ^11^ Department of Clinical Research Phoenix Children's Hospital Phoenix Arizona USA; ^12^ Division of Pediatric Gastroenterology Phoenix Children's Hospital Phoenix Arizona USA

**Keywords:** Crohn's disease, payor barriers, prior authorization, treatment delay and denial, ulcerative colitis

## Abstract

**Objectives:**

Early biologic initiation, dose optimization, and therapy modification based on disease phenotype are key to improving outcomes in pediatric inflammatory bowel disease (IBD). Enacting optimized therapy is often impeded by the lack of United States Food and Drug Administration (FDA) approval for pediatric use of newer advanced therapies or intensified dosing regimens. These barriers often result in initial payor denial of coverage and added prior authorization burden on physicians, leading to patient delays in medication initiation and therapy optimization, and development of disease‐related morbidity.

**Methods:**

A sample of pediatric patients experiencing payor barriers to IBD biologic treatment, containing data on treatment delays and adverse outcomes, was obtained through a nationwide survey of pediatric gastroenterology providers via a longstanding, widely used pediatric gastroenterology Listserv (housed at University of Vermont) from January 2023 to August 2023.

**Results:**

Providers across the United States reported information for 113 patients experiencing payor barriers for biologics IBD treatment. Ultimately, 77% of initial denials were approved. The median time to receiving medication was 18 days, with administrative time (prior authorization and appeal) requiring a median of 180 min. More than half (60%) of patients experienced adverse outcomes or worsened quality of life due to delays in treatment, including 21% of patients who were hospitalized.

**Conclusions:**

These findings highlight the detrimental impact of payor barriers to treatment for children with IBD. Reforms that minimize delays in care and provider administrative burden are imperative to ensure that children receive timely evidence‐based treatment that improves disease outcomes and prevents adverse events.

## Abstract (highlights)

**What is Known:**

- Prior authorization, mandated “step therapy,” and formulary restrictions increase treatment delays, healthcare utilization, and administrative burden in managing inflammatory bowel disease (IBD).
- Pediatric patients are more likely to experience treatment delays/denials due to the lack of federally‐approved therapies.

**What is New:**

- Prescribed medications, payor organizations and encountered barrier to pediatric IBD treatment are presented.
- Barriers to treatment by payors, of which 77% are ultimately approved, result in substantive treatment delay, patient harm, and hospitalization.

## 1 INTRODUCTION

Biologic and small molecule therapies have revolutionized the treatment of pediatric inflammatory bowel disease (IBD) through their associations with improved quality of life, growth, and remission rates, as well as with decreased disease complications, hospitalization, and surgeries.^1^, ^2^, ^3^, ^4^, ^5^ Mounting evidence has further demonstrated the importance of early biologic initiation, as well as therapeutic drug monitoring for dose escalation and modification, particularly when children have inadequate or poor responses to initial dosing.^5^, ^6^, ^7^, ^8^, ^9^, ^10^, ^11^, ^12^, ^13^ Current pediatric IBD guidelines call for early initiation of biologics as well as therapeutic drug monitoring.^3^, ^14^, ^15^, ^16^ Nevertheless, insurers and pharmacy benefit managers (PBMs) have incorporated various policies, including mandated step therapy, which can delay such care. Step therapy is a utilization‐management strategy where insurers require patients to trial a series of treatment pathways for various conditions before approving the initially‐prescribed restricted agent.^17^ Notably, while designed to improve quality and costs of care, studies have shown these strategies to be less effective or associated with increased risk of adverse events.^18^, ^19^, ^20^

Treatment delays in pediatric IBD have been associated with disease progression, hospitalizations, surgeries, and preventable corticosteroid dependence.^18^, ^19^, ^20^, ^21^, ^22^, ^23^, ^24^ While at least 10 biologics or small molecules are available for treatment of adult IBD, only infliximab and adalimumab are currently approved by the United States Food and Drug Administration (FDA) for use in pediatric patients. Off‐label use of many such medications is often associated with payor barriers, such as prior authorization, that can be detrimental to patients and health systems. Notably, there remains scarce research regarding the effects of payor barriers on treatment delay for pediatric patients with IBD and added administrative workload. To better characterize this experience, we conducted a national survey eliciting provider experience with payor barriers to pediatric IBD therapies. Our aim was to document payor barriers encountered, the final payor coverage decision, as well as potentially avoidable consequences including treatment delays in care, patient morbidity and administrative burden, across community and academic pediatric gastroenterology practices.

## 2 METHODS

We conducted a nationwide survey through a widely utilized pediatric gastroenterology Listserv (Pediatric Bulletin Board, housed at University of Vermont; pedgi@list.uvm.edu) from January 2023 to August 2023 using the Research Electronic Data Capture system (REDCap; Vanderbilt). This Listserv is comprised of pediatric gastroenterologists, trainees (fellows and residents), and advanced practice providers. Notably, this was inclusive of pediatric gastrointestinal (GI) providers; at the time of the survey, there were approximately 2800 pediatric GI providers in the United States. Listserv members were surveyed regarding their experience with payor barriers to pediatric IBD treatment and requested to provide patient‐specific examples, including data on demographics (age, gender, location), medication prescribed, payor information (insurance/PBM, barrier/reason for initial denial or delay, alternative recommendation), authorization outcomes (time to receiving medication, administrative time, final approval), and patient outcomes (bridge therapy, patient harm, and hospitalization rates). The full survey questionnaire is included in the Supplemental materials. Providers anonymously submitted survey information. The payor Blue Cross Blue Shield (BCBS) can vary state‐by‐state, but was categorized as a single entity for descriptive analyses. Administrative time was defined as staff time devoted to phone calls and paperwork related to a claim. Patient harm was defined as the physician's perception of a deleterious effect on quality of life, requirement of transfusion, surgery, or hospitalization for their patient.

### 2.1 Statistical analysis

Descriptive statistics summarized patient demographics, prescribed medications, characteristics related to payors, and patient outcomes. Bivariate analyses examined the relationship between characteristics (patient age and gender, medication, payor, barrier/reason for treatment delay/denial, any alternate recommendation) and outcomes (final approval, time to treatment, bridge therapy, patient harm, hospitalizations, and administrative time). Association analyses employed the Fisher's exact, Wilcoxon rank sum, or Kruskal Wallis tests, as appropriate. Patients whose treatment was not ultimately approved were excluded from statistical analysis of treatment delay and administrative time, to avoid bias in these estimates. All statistical tests were two‐sided and evaluated at the 0.05 significance level. Statistical analyses were performed using SAS software (Statistical Analysis Software 9.4; SAS Institute Inc.).

### 2.2 Ethical consideration

This study was determined to be a quality improvement project and not human subjects research by the Phoenix Children's Hospital ethics review board.

## 3 RESULTS

### 3.1 Demographics

Of the 171 reported patient cases, 58 (34%) were excluded [33 ages ≥ 18 years, 21 non‐IBD, 2 requests for Flagyl/Azithromycin, and 2 requests for 5‐aminosalicylic acid (5‐ASA)]. Study subjects included the remaining 113 pediatric patients, reported by providers from 30 US states, with most reported from Ohio (*N* = 27, 24%) and Arizona (*N* = 8, 7%) (Supporting Information S1: Figure S1). The median (interquartile range [IQR]) age of all pediatric patients cared for by respondent providers was 14 (11–16) years, with 59 (52%) females and 54 (48%) males (Table 1).

**Table 1:** Association of patient characteristics, payor barriers and outcomes with medications prescribed for inflammatory bowel disease (IBD) in pediatric patients.

Factor	Total patients (*N* = 113)	Medications						
		Infliximab/inflectra/avsola (*N* = 42)	Adalimumab (*N* = 31)	Ustekinemab (*N* = 27)	Vedolizumab (*N* = 6)	Risankizumab (*N* = 1)	Tofacitinib/upacitinib (*N* = 6)	*p* Value
Age (years)								0.63^a^
Mean (SD)	13.0 (3.5)	12.9 (3.7)	12.4 (4.0)	13.4 (2.8)	14.8 (1.5)	11.0 (–)	14.0 (4.4)	
Median (IQR)	14 (11, 16)	14 (10, 16)	13 (11, 16)	14 (11, 16)	14.5 (14, 16)	11 (11, 11)	16.5 (10, 17)	
Sex, *N* (%)								0.94^2^
Female	59 (52.2)	23 (54.8)	16 (51.6)	13 (48.1)	3 (50.0)	0 (0.0)	4 (66.7)	
Male	54 (47.8)	19 (45.2)	15 (48.4)	14 (51.9)	3 (50.0)	1 (100.0)	2 (33.3)	
Insurance vs. PBM, *N* (%)								<0.0001^b^
Insurance	95 (84.1)	41 (97.6)	18 (58.1)	24 (88.9)	6 (100.0)	0 (0.0)	6 (100.0)	
PBM	18 (15.9)	1 (2.4)	13 (41.9)	3 (11.1)	0 (0.0)	1 (100.0)	0 (0.0)	
Alternative recommendation, *N* (%) (*missing* = 26)								0.11^b^
No	67 (77.0)	22 (68.8)	14 (70.0)	22 (95.7)	4 (80.0)	1 (100.0)	4 (66.7)	
Yes	20 (23.0)	10 (31.3)	6 (30.0)	1 (4.3)	1 (20.0)	0 (0.0)	2 (33.3)	
Prior authorization, *N* (%)								0.50^b^
No	21 (18.6)	8 (19.0)	3 (9.7)	8 (29.6)	1 (16.7)	0 (0.0)	1 (16.7)	
Yes	92 (81.4)	34 (81.0)	28 (90.3)	19 (70.4)	5 (83.3)	1 (100.0)	5 (83.3)	
Dose restriction, *N* (%)								0.02^b^
No	71 (62.8)	19 (45.2)	24 (77.4)	17 (63.0)	4 (66.7)	1 (100.0)	6 (100.0)	
Yes	42 (37.2)	23 (54.8)	7 (22.6)	10 (37.0)	2 (33.3)	0 (0.0)	0 (0.0)	
Step therapy, *N* (%)								0.008^b^
No	94 (83.2)	37 (88.1)	19 (61.3)	26 (96.3)	5 (83.3)	1 (100.0)	6 (100.0)	
Yes	19 (16.8)	5 (11.9)	12 (38.7)	1 (3.7)	1 (16.7)	0 (0.0)	0 (0.0)	
Formulary/nonmedical switch, *N* (%)								0.04^b^
No	101 (89.4)	34 (81.0)	29 (93.5)	27 (100.0)	6 (100.0)	1 (100.0)	4 (66.7)	
Yes	12 (10.6)	8 (19.0)	2 (6.5)	0 (0.0)	0 (0.0)	0 (0.0)	2 (33.3)	
Final approval, *N* (%) (*missing* = 9)								0.30^b^
No	24 (23.1)	6 (14.6)	7 (23.3)	8 (34.8)	1 (20.0)	0 (0.0)	2 (40.0)	
Yes	80 (76.9)	35 (85.4)	23 (76.7)	15 (65.2)	4 (80.0)	0 (0.0)	3 (60.0)	
**Delay** (days) (*missing* = 38)								0.37^a^
Mean (SD)	29.0 (37.4)	35.2 (49.9)	24.1 (28.8)	27.4 (18.7)	26.8 (22.8)	–	7.3 (6.4)	
Median (IQR)	18 (10, 35)	21 (10, 37.5)	14 (10, 28)	28 (10, 40)	18.5 (12, 41.5)	–	10 (0, 12)	
Bridge therapy, *N* (%) (*missing* = 4)								0.02^b^
No	57 (52.3)	14 (34.1)	20 (69.0)	18 (66.7)	2 (33.3)	0 (0.0)	3 (50.0)	
Yes	52 (47.7)	27 (65.9)	9 (31.0)	9 (33.3)	4 (66.7)	0 (0.0)	3 (50.0)	
Patient harm, *N* (%) (*missing* = 4)								0.74^b^
No	44 (40.4)	15 (35.7)	15 (48.4)	11 (45.8)	1 (20.0)	0 (0.0)	2 (33.3)	
Yes	65 (59.6)	27 (64.3)	16 (51.6)	13 (54.2)	4 (80.0)	1 (100.0)	4 (66.7)	
Hospitalization, *N* (%) (*missing* = 4)								0.08^b^
No	86 (78.9)	30 (71.4)	29 (93.5)	17 (70.8)	5 (100.0)	1 (100.0)	4 (66.7)	
Yes	23 (21.1)	12 (28.6)	2 (6.5)	7 (29.2)	0 (0.0)	0 (0.0)	2 (33.3)	
Administrative time (min) (*missing* = 37)								0.48^a^
Mean (SD)	288 (385)	386 (521)	159 (94)	263 (267)	242.5 (240)	–	253 (301)	
Median (IQR)	180 (100, 300)	190 (120, 360)	120 (90, 180)	165 (90, 300)	140 (105, 380)	–	100 (60, 600)	

Abbreviations: IQR, interquartile range; PBM, pharmacy benefit manager; SD, standard deviation.

^a^ Kruskal–Wallis test compared the medians of each quantitative factor between medications.

^b^ Fisher's exact test compared the level proportions of each categorical factor between medications.

### 3.2 Medications

Medications requested included infliximab (or biosimilar) for 42 (37%) patients, adalimumab for 31 (27%), ustekinumab for 27 (24%) patients, with fewer requests of vedolizumab for 6 (5.3%), tofacititinib/upadacitinib for 6 (5.3%), and risankizumab for 1 (0.9%) patient (Table 1, Supporting Information S1: Figure S2). Medication was significantly associated (*p* < 0.0001) with payor being an insurance company versus PBM (Table 1).

### 3.3 Payors

**Figure 1:**
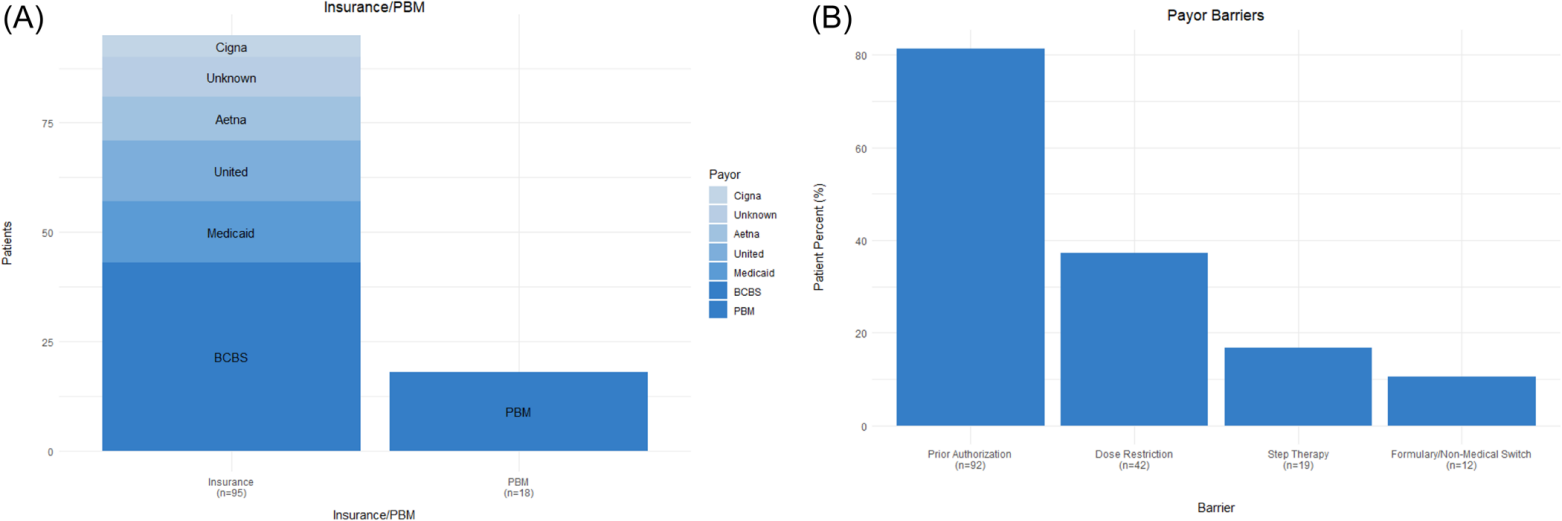
(A) Frequency of pediatric patients with inflammatory bowel disease (IBD) prescribed biologics medications by payor. (B) Payor barriers to treatment with biologics medications for pediatric patients with IBD. PBM = pharmacy benefit manager; BCBS = Blue Cross Blue Shield.

There were six payors, 5 insurance companies and 1 PBM, providing coverage to 95 (84%) and 18 (16%) patients, respectively (Figure 1A, Supporting Information S1: Table S1).

### 3.4 Barriers to treatment

Prior authorization was the most frequent reason cited for treatment delay/denial in 92 (81%) patients, with dose restrictions in 42 (37%), mandated “step therapy” policies in 19 (17%), and required non‐FDA approved nonmedical switches/formulary issues in 12 (11%) patients (Figure 1B, Table 1). Dose‐restriction as the reason for delay/denial was significantly associated (*p* = 0.02) with medication; cited most frequently for Infliximab or its biosimilar in 23 (55%) patients, Ustekinumab in 10 (37%), Vedolizumab in 2 (33%) and Adalimumab in 7 (23%) patients (Table 1). Significant associations with medications were also detected for treatment delay/denials due to “step therapy” (*p* = 0.008) and formulary issues (*p* = 0.04); however, prior authorization as a reason for treatment delay/denial was not associated with medication (Table 1).

### 3.5 Final approval of coverage by payor

Most patients, 80 (77%) ultimately received approval for the medication or dosing initially requested (Table 1). The rate of final approval was higher for payors requesting prior authorization vs other barriers/reasons (83% vs. 50%; *p* = 0.003) (Supporting Information S1: Table S2). No association was detected between medication or insurance/PBM with final approval (Table 2).

**Table 2:** Association of patient characteristics and payor barriers with final approval, bridge therapy, patient harm, and hospitalization.

Factor	Total patients (*N* = 113)				Outcome								
		Final approval			Bridge therapy			Patient harm			Hospitalization		
		No (*N* = 24)	Yes (*N* = 80)	*p* Value	No (*N* = 57)	Yes (*N* = 52)	*p* Value	No (*N* = 44)	Yes (*N* = 65)	*p* Value	No (*N* = 86)	Yes (*N* = 23)	*p* Value
Age (years)				0.24^a^			0.27^a^			0.08^a^			0.31^a^
Mean (SD)	13.0 (3.5)	15 (3.3)	14 (4.2)		13.4 (3.6)	12.9 (3.4)		13.8 (3.3)	12.5 (3.7)		13.2 (3.6)	12.4 (3.6)	
Median (IQR)	14 (11, 16)	14.5 (11.5, 16)	13 (10, 16)		15 (11, 16)	13 (11, 16)		15 (11.5, 16.5)	13 (10, 16)		14 (11, 16)	12 (10, 16)	
Sex, *N* (%)				1.00^b^			0.08^b^			0.05^b^			0.03^b^
Female	59 (52.2)	13 (54.2)	42 (52.5)		26 (45.6)	33 (63.5)		18 (40.9)	40 (61.5)		41 (47.7)	17 (73.9)	
Male	54 (47.8)	11 (45.8)	38 (47.5)		31 (54.4)	19 (36.5)		26 (59.1)	25 (38.5)		45 (52.3)	6 (26.1)	
Medication requested, *N* (%)				0.30^b^			0.02^b^			0.74^b^			0.08^b^
Infliximab/inflectra/avsola	42 (37.2)	6 (25.0)	35 (43.8)		14 (24.6)	27 (51.9)		15 (34.1)	27 (41.5)		30 (34.9)	12 (52.2)	
Adalimumab	31 (27.4)	7 (29.2)	23 (28.8)		20 (35.1)	9 (17.3)		15 (34.1)	16 (24.6)		29 (33.7)	2 (8.7)	
Ustekinemab	27 (23.9)	8 (33.3)	15 (18.8)		18 (31.6)	9 (17.3)		11 (25.0)	13 (20.0)		17 (19.8)	7 (30.4)	
Vedolizumab	6 (5.3)	1 (4.2)	4 (5.0)		2 (3.5)	4 (7.7)		1 (2.3)	4 (6.2)		5 (5.8)	0 (0.0)	
Tofacitinib/upacitinib	6 (5.3)	2 (8.3)	3 (3.8)		3 (5.3)	3 (5.8)		0 (0.0)	1 (1.5)		1 (1.2)	0 (0.0)	
Risankizumab	1 (0.9)	–	–		–	–		2 (4.5)	4 (6.2)		4 (4.7)	2 (8.7)	
Insurance vs. PBM, *N* (%)				0.35^b^			0.002^b^			0.02^b^			0.01^b^
Insurance	95 (84.1)	22 (91.7)	65 (81.3)		43 (75.4)	50 (96.2)		32 (72.7)	59 (90.8)		68 (79.1)	23 (100.0)	
PBM	18 (15.9)	2 (8.3)	15 (18.8)		14 (24.6)	2 (3.8)		12 (27.3)	6 (9.2)		18 (20.9)	0 (0.0)	
Alternative recommendation, *N* (%) (*missing* = 26)				0.38^b^			0.30^b^			0.80^b^			0.55^b^
No	67 (77.0)	16 (84.2)	43 (72.9)		33 (82.5)	31 (72.1)		29 (78.4)	34 (73.9)		49 (77.8)	14 (70.0)	
Yes	20 (23.0)	3 (15.8)	16 (27.1)		7 (17.5)	12 (27.9)		8 (21.6)	12 (26.1)		14 (22.2)	6 (30.0)	
Prior authorization, *N* (%)				0.003^b^			0.47^b^			0.80^b^			1.00^b^
No	21 (18.6)	10 (41.7)	10 (12.5)		9 (15.8)	12 (23.1)		7 (15.9)	12 (18.5)		15 (17.4)	4 (17.4)	
Yes	92 (81.4)	14 (58.3)	70 (87.5)		48 (84.2)	40 (76.9)		37 (84.1)	53 (81.5)		71 (82.6)	19 (82.6)	
Dose restriction, *N* (%)				0.16^b^			0.55^b^			0.23^b^			0.81^b^
No	71 (62.8)	12 (50.0)	53 (66.3)		37 (64.9)	30 (57.7)		31 (70.5)	38 (58.5)		55 (64.0)	14 (60.9)	
Yes	42 (37.2)	12 (50.0)	27 (33.8)		20 (35.1)	22 (42.3)		13 (29.5)	27 (41.5)		31 (36.0)	9 (39.1)	
Step therapy, *N* (%)				0.76^b^			0.10^b^			0.30^b^			0.35^b^
No	94 (83.2)	21 (87.5)	65 (81.3)		52 (91.2%)	41 (78.8)		34 (77.3)	56 (86.2)		69 (80.2)	21 (91.3)	
Yes	19 (16.8)	3 (12.5)	15 (18.8)		5 (8.8%)	11 (21.2)		10 (22.7)	9 (13.8)		17 (19.8)	2 (8.7)	
Formulary/nonmedical switch, *N* (%)				1.00^b^			0.55^b^			0.76^b^			0.71^b^
No	101 (89.4)	22 (91.7)	71 (88.8)		52 (91.2)	45 (86.5)		40 (90.9)	57 (87.7)		77 (89.5)	20 (87.0)	
Yes	12 (10.6)	2 (8.3)	9 (11.3)		5 (8.8)	7 (13.5)		4 (9.1)	8 (12.3)		9 (10.5)	3 (13.0)	
Final approval, *N* (%) (*missing* = 9)				–			1.00^2^			0.63^b^			0.57^b^
No	24 (23.1)	–	–		13 (24.5)	11 (22.9)		8 (19.0)	15 (24.6)		17 (21.0)	6 (27.3)	
Yes	80 (76.9)	–	–		40 (75.5)	37 (77.1)		34 (81.0)	46 (75.4)		64 (79.0)	16 (72.7)	
Delay (days) (*missing* = 38)				–			0.0003^a^			<0.0001^a^			0.10^a^
Mean (SD)	29.0 (37.4)	–	29.0 (37.4)		20.1 (25.9)	40.2 (45.5)		12.7 (13.3)	40.4 (44.3)		28.1 (40.0)	32.8 (23.8)	
Median (IQR)	18 (10, 35)	–	18 (10, 35)		12 (7, 21)	28 (19, 50)		10 (4, 15)	28 (14, 53.5)		14 (10, 28)	27.5 (14, 40)	
Bridge therapy, *N* (%) (*missing* = 4)				1.00^b^			–			<0.0001^b^			0.002^b^
No	57 (52.3)	13 (54.2)	40 (51.9)		–	–		32 (78.0)	22 (34.4)		49 (59.8)	5 (21.7)	
Yes	52 (47.7)	11 (45.8)	37 (48.1)		–	–		9 (22.0)	42 (65.6)		33 (40.2)	18 (78.3)	
Patient harm, *N* (%) (*missing* = 4)				0.63^b^			<0.0001^b^			–			<0.0001^b^
No	44 (40.4)	8 (34.8)	34 (42.5)		32 (59.3)	9 (17.6)		–	–		44 (51.2)	0 (0.0)	
Yes	65 (59.6)	15 (65.2)	46 (57.5)		22 (40.7)	42 (82.4)		–	–		42 (48.8)	23 (100.0)	
Hospitalization, *N* (%) (*missing* = 4)				0.57^b^			0.002^b^			<0.0001^b^			–
No	86 (78.9)	17 (73.9)	64 (80.0)		49 (90.7)	33 (64.7)		44 (100.0)	42 (64.6)		–	–	
Yes	23 (21.1)	6 (26.1)	16 (20.0)		5 (9.3)	18 (35.3)		0 (0.0)	23 (35.4)		–	–	
Administrative time (min) (*missing* = 37)				–			0.0004^a^			0.01^a^			0.02^a^
Mean (SD)	288 (385)	–	288 (385)		179 (185)	430 (510)		195 (265)	359 (447)		276 (419)	335 (201)	
Median (IQR)	180 (100, 300)	–	180 (100, 300)		120 (90, 180)	300 (180, 480)		120 (90, 180)	240 (100, 360)		120 (90, 240)	300 (200, 600)	

Abbreviations: IQR, interquartile range; PBM, pharmacy benefit manager; SD, standard deviation.

^a^ Wilcoxon rank sum test compared the medians of each quantitative factor between levels of each outcome.

^b^ Fisher exact test compared the level proportions of each categorical factor between levels of each outcome.

### 3.6 Delay to treatment

**Figure 2:**
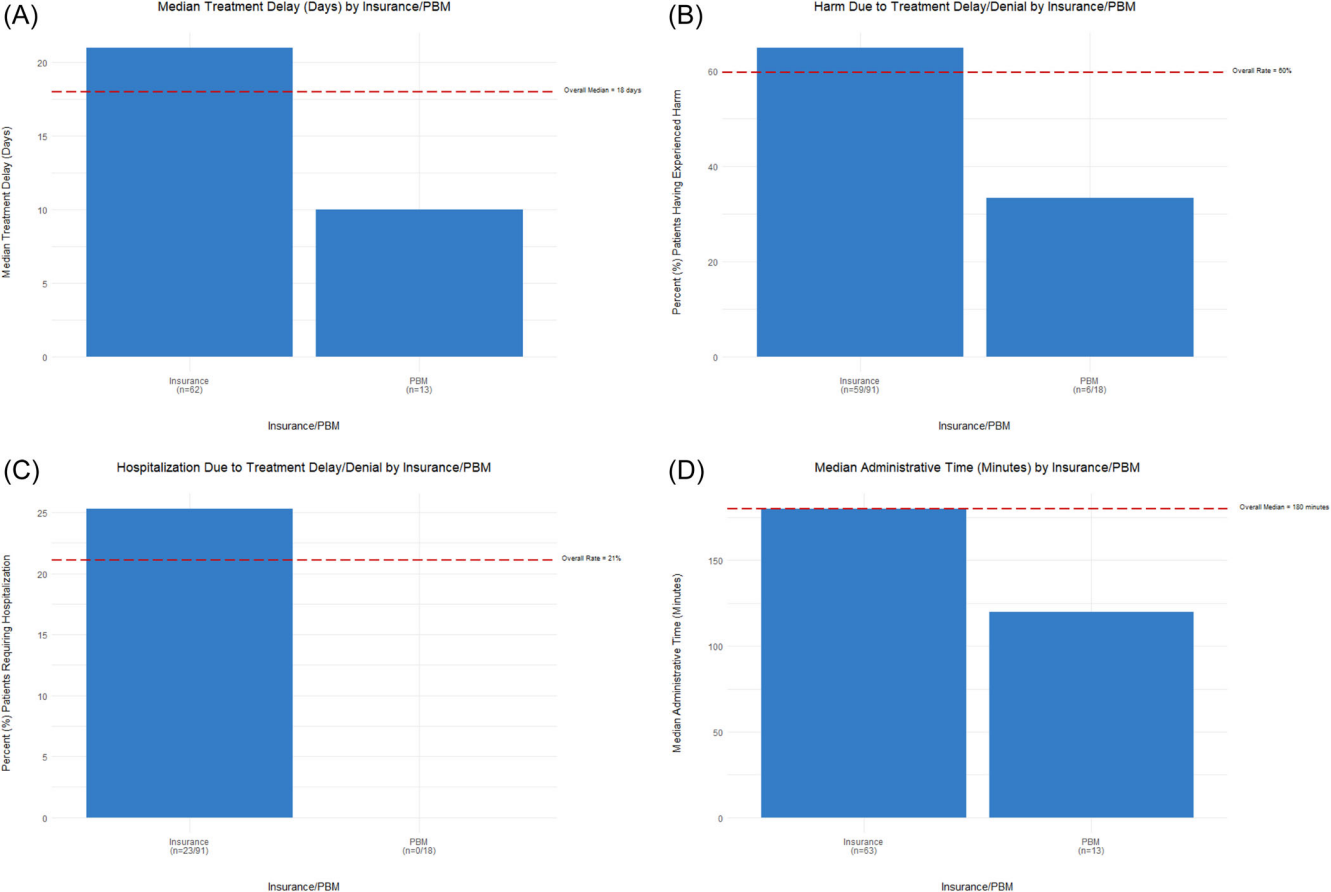
(A) Median delay time (days) to treatment with biologics medications for pediatric patients with inflammatory bowel disease (IBD) by payor. Also shown are percentages of pediatric patients with IBD experiencing (B) harm and (C) hospitalization due to treatment delay/denial with biologics medications by payor. (D) Median administrative time (minutes) required to respond to payor barriers to treatment with biologics medications for pediatric patients with IBD by payor. PBM, pharmacy benefit manager.

The median (IQR) delay time to receipt of the prescribed medication was 18 (10, 35) days (Table 1). Patients with coverage from insurance vs PBM payors had a significantly longer median delay time to receipt of prescribed medication (21 vs. 10 days; *p* = 0.02) (Figure 2A, Supporting Information S1: Table S1). The prescribed medication was not associated with treatment delay (*p* = 0.37) (Table 1). To view median delay to treatment by medication type, please see Supporting Information S1: Figure S3.

### 3.7 Bridge therapy, patient harm, and hospitalization

During the appeal process, 52 (48%) patients required an alternate bridging therapy (Table 2). Patient harm, defined by physician's perception of a deleterious effect on the patient's quality of life, requirement of transfusion, surgery, or hospitalization, was reported in 65 (60%) cases, with 23 (21%) of these involving hospitalization (Table 2). Medication was significantly associated with bridge therapy (*p* = 0.02), but no association was detected with harm (*p* = 0.74) or hospitalization (*p* = 0.08) (Table 2). Comparisons of insurance vs PBM detected significantly higher rates of bridge therapy (54% vs 12%, *p* = 0.002), patient harm (65% vs. 33%, *p* = 0.02) and hospitalization (25% vs 0%; *p* = 0.01) for patients with insurance coverage (Supporting Information S1: Table S1, Figure 2B,C). To view percent (%) patient harm and hospitalization by medication type, please see Supporting Information S1: Figures S4 and S5.

### 3.8 Administrative time

The overall median (IQR) administrative time spent to address payor barriers was 180 (100, 300) min (Figure 2D, Table 1). Significantly longer administrative time was detected between insurance versus PBM (180 vs. 120 min; *p* = 0.04) (Supporting Information S1: Table S1). Median administration time addressing payor barriers (in days) by medication type is demonstrated in Supporting Information S1: Figure S6. There was no association between payor barriers (prior authorization, dose restriction, step therapy, formulary/nonmedical switch) and amount of administrative time (Supporting Information S1: Table S2).

## 4 DISCUSSION

Findings of this study highlight the detrimental impact of payor (insurance/PBM) barriers to physician prescribed treatment on children with IBD. The care of pediatric patients with Crohn's disease and ulcerative colitis is different from the adult population, where children often experience more aggressive disease, growth failure, and harm associated with systemic corticosteroid treatment. Despite federal regulatory and legislative attempts to expedite the pediatric drug review process, FDA approval of medications for pediatric patients usually lags behind adult medication approvals by approximately 8–10 years; such FDA delays have increasingly resulted in denial of coverage by payors.^19^

In our pediatric IBD population, prior authorization for a biologic or small molecule medication can delay/deny treatment due to off‐label use of the requested medication or available evidence‐based guidelines.^19^ Prescriptions of larger doses or more frequent dosing intervals than is federally approved for pediatric IBD may also be denied by payors. An initial denial can result in a sequence of multiple appeals, with limited regulation or rules specifying the time required for payor responses. In addition to delayed patient care, payor appeal processes increase provider administrative burden and decrease satisfaction, leading to moral distress and burnout.^25^, ^26^

Another payor barrier to coverage for appropriate treatment is step therapy. This requirement usually specifies a certain amount of time, up to several months, that an agent must be tried to document failure. Thirty percent of policies require the failure of at least two agents before approval.^27^ However, a recent review of insurance company requests for step therapy found many inconsistencies with currently published practice guidelines.^28^, ^29^

Our results show that payor barriers to coverage were overcome, and treatment was ultimately approved for most (77%) patients, highlighting the preventable nature of patient harms where most payors ultimately concur with providers. These barriers are also inconsistent across payors; indeed, a recent study from Connecticut revealed private insurance took 9.1 days longer for biologic initiation compared to Medicaid for children with IBD.^30^

In this study, prescribed treatment delay/denial resulted in substantial patient harms, with 60% of patients experiencing an adverse outcome, and 21% of patients requiring hospitalization. This is a national and public health concern, as well as a financial burden on health systems and families. Constant, et al. found similar results, with a median delay in treatment of 10–25 days due to prior authorization, “step therapy,” and peer‐to‐peer review process, ultimately resulting in an increase of healthcare utilization within 180 days by 12.9%.^18^

Numerous literature reviews have illustrated the harmful effects of formulary restrictions and prior authorization on patient outcomes, health resource utilization, and cost of care due to treatment delays.^24^, ^31^ A recent survey of adult and pediatric gastroenterologists indicated that 83% of providers reported an adverse outcome due to prior authorization‐related biologic delay. They also found that 97% of providers felt the prior authorization process worsened quality of care and, equally concerning, 97% listed this as a key contributor in physician burnout.^32^ The American Medical Association recently performed a survey that found more than one‐third of physicians believe prior authorizations have led to serious adverse effects in their patients. Another survey by the Crohn's and Colitis Foundation revealed significant concerns over cost, payor denials, and delayed access to care. In pediatrics, this burden has a bystander effect on families, which spills over to impact employment, treatment choices, and overall quality of life.^33^, ^34^ A survey of adult care providers in 2018 found that physicians were completing a mean of 31 prior authorizations per week, costing an average of 15 h of administrative time. A more recent study showed physicians spent a median of 4 h/week, with nurses spending 15 h/week and other staff spending up to 10 h/week, on drug utilization management. The value of this time was estimated to be $75,927 per physician per year, extrapolated to more than $43 billion nationally.^35^

There are notable limitations to this study due to data collection via a provider survey, including recall bias and selection bias. Indeed, providers with strong negative experiences regarding payor barriers to care were likely more compelled to respond, and also had likely greater recall for specific cases. Future work would benefit from collecting information through more unbiased methods, such as a large chart review or a prospective multicenter cohort study. Without specific reference points for variables such as diminished quality of life or time to medication receipt, providers made their own judgments in providing survey responses; this likely decreased response accuracy. To more objectively assess outcome data, future studies would define and utilize consistent cutoffs and reference points. Surveys were completed anonymously, with multiple responses potentially submitted by the same provider and multiple providers from the same institution; this could have reduced the generalizability of the current findings. While our response rate appears low, it should be noted that only a proportion of Listserv participants provide routine IBD care. In this regard, our response rate likely underestimates the impact of common insurance industry practices on the child with IBD, their family, their providers, and the pediatric healthcare system. Notably, the current project was unable to distinguish outcomes by types of IBD (i.e., ulcerative colitis, Crohn's disease, etc.); future work should explore whether payor barriers resulting in treatment delays/denials differ by disease type. In addition, some surveys were completed before knowing the final payor decision, leading to missing outcome status. To provide a more unbiased estimate of rates for barriers to treatment by payor, future work could collect and account for market shares for patients with IBD.

Despite stated limitations, this study identified the widespread practice of payor barriers to coverage and delay of therapy in children with IBD, leading to patient harms including unnecessary exposure to steroids, hospitalization, and surgery. In addition to detrimental patient outcomes, these barriers have the potential to interfere with informed medical decision making, hinder the physician‐patient relationship, and place an administrative burden on healthcare providers. While the nature of the current payor procedures is undoubtedly complex, further research to inform policy changes is crucial to minimize unnecessary and avoidable harm, and achieve optimal care and health outcomes for pediatric patients with IBD.

## CONFLICT OF INTEREST STATEMENT

The authors declare no conflicts of interest.

## Supporting information

Supporting information.
