# Supplementary material for: National perspectives of barriers by insurance and pharmacy benefit managers in pediatric inflammatory bowel disease
Source: JPGN Rep. 2025 Feb 10;6(2):80–90. doi: 10.1002/jpr3.70004 (PMC12078066; doi:10.1002/jpr3.70004)
Supplement: Supplementary file 1 — Supporting information. [file JPR3-6-80-s001.docx]

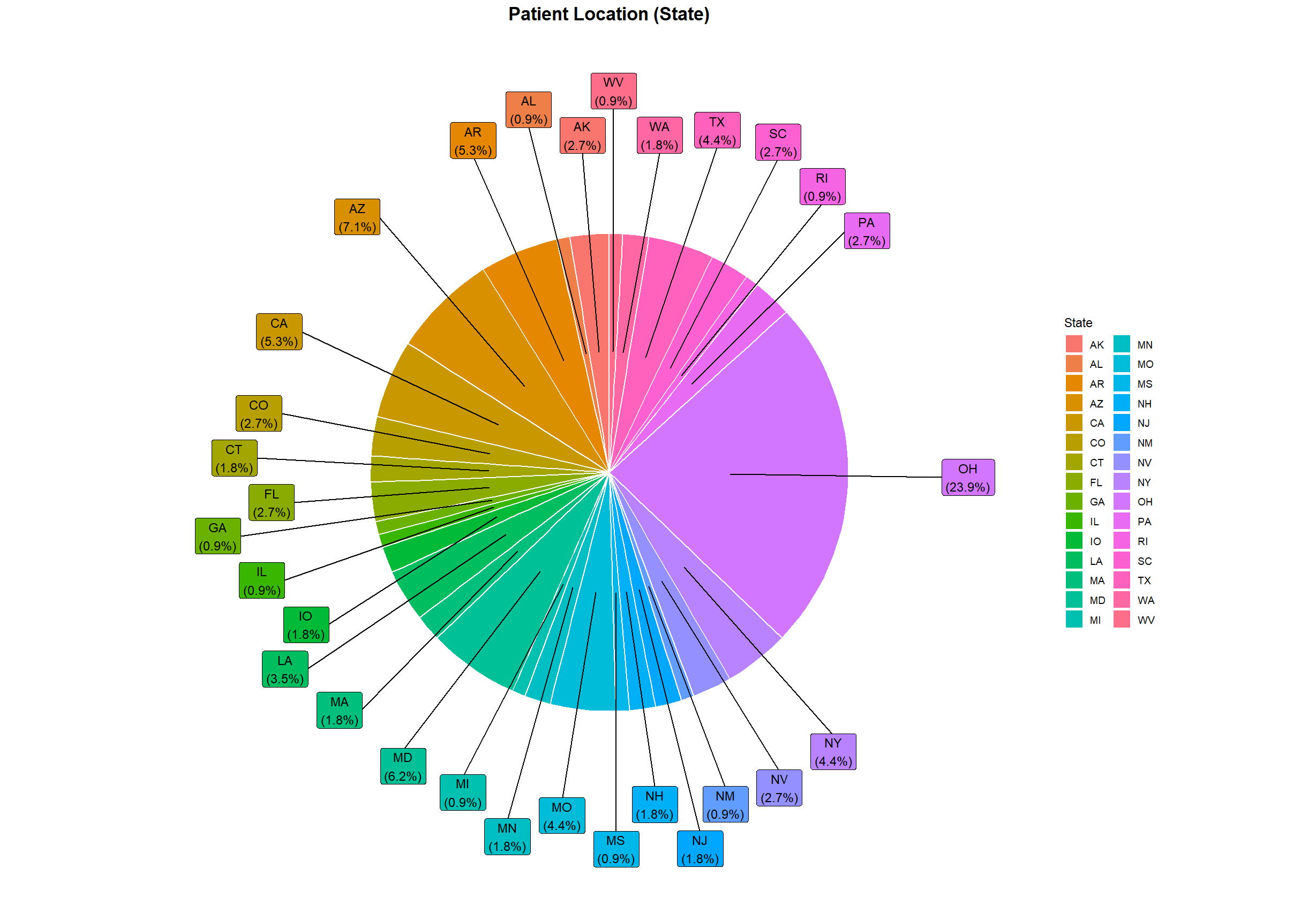


**Figure S1:** Pediatric patients with inflammatory bowel disease (IBD) facing payor barriers to treatment with biologics medications reported from each state within the United States (US).


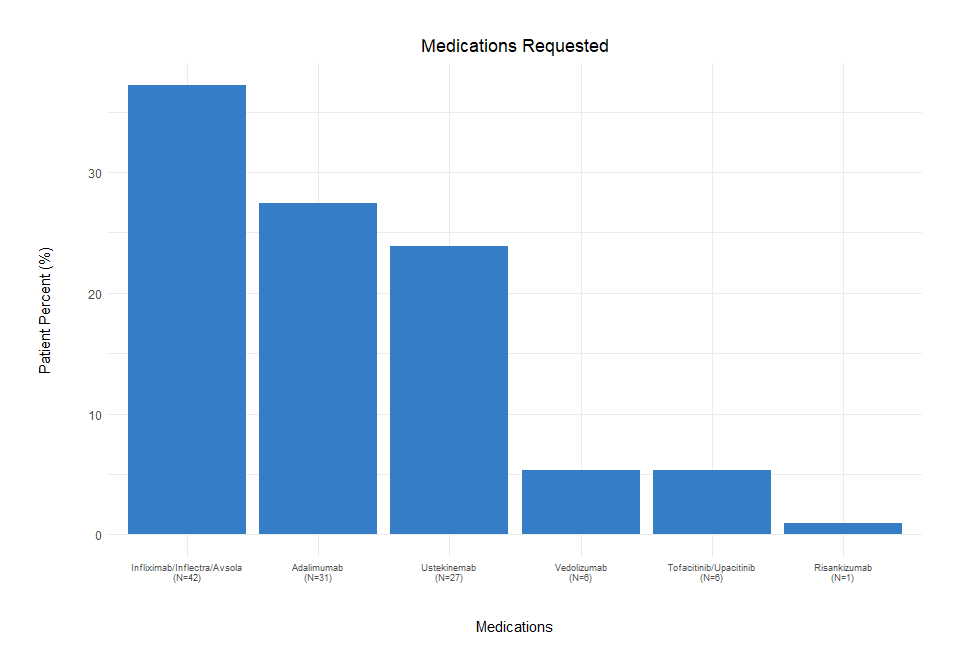


**Figure S2:** Pediatric patients with inflammatory bowel disease (IBD) experiencing delay/denial of treatment with biologics medications.

**Table S1:** Association of patient characteristics, prescribed medications, encountered barriers and outcomes with payors.

| **Factor** | Total Patients (N=113) | **Payors** | | |
| --- | --- | --- | --- | --- |
|  |  | Insurance  (N=95) | PBM  (N=18) | P-value |
| **Age** (years) |  |  |  | 0.60^1^ |
| Mean (SD) | 13.0 (3.5) | 13.0 (3.5) | 13.3 (3.6) |  |
| Median (IQR) | 14 (11, 16) | 13 (11, 16) | 14 (11, 17) |  |
|  |  |  |  |  |
| **Sex**, N (%) |  |  |  | 0.61^2^ |
| Female | 59 (52.2%) | 51 (53.7%) | 8 (44.4%) |  |
| Male | 54 (47.8%) | 44 (46.3%) | 10 (55.6%) |  |
|  |  |  |  |  |
| **Medication Requested**, N (%) |  |  |  | <0.0001^2^ |
| Infliximab/Inflectra/Avsola | 42 (37.2%) | 41 (43.2%) | 1 (5.6%) |  |
| Adalimumab | 31 (27.4%) | 18 (18.9%) | 13 (72.2%) |  |
| Ustekinemab | 27 (23.9%) | 24 (25.3%) | 3 (16.7%) |  |
| Vedolizumab | 6 (5.3%) | 6 (6.3%) | 0 (0.0%) |  |
| Risankizumab | 1 (0.9%) | 0 (0.0%) | 1 (5.6%) |  |
| Tofacitinib/Upacitinib | 6 (5.3%) | 6 (6.3%) | 0 (0.0%) |  |
|  |  |  |  |  |
| **Alternative Recommendation**, N (%)  *(Missing = 26)* |  |  |  | 1.00^2^ |
| No | 67 (77.0%) | 59 (76.6%) | 8 (80.0%) |  |
| Yes | 20 (23.0%) | 18 (23.4%) | 2 (20.0%) |  |
|  |  |  |  |  |
| **Prior Authorization**, N (%) |  |  |  | 0.02^2^ |
| No | 21 (18.6%) | 21 (22.1%) | 0 (0.0%) |  |
| Yes | 92 (81.4%) | 74 (77.9%) | 18 (100.0%) |  |
|  |  |  |  |  |
| **Dose Restriction**, N (%) |  |  |  | 0.80^2^ |
| No | 71 (62.8%) | 59 (62.1%) | 12 (66.7%) |  |
| Yes | 42 (37.2%) | 36 (37.9%) | 6 (33.3%) |  |
|  |  |  |  |  |
| **Step Therapy**, N (%) |  |  |  | 0.73^2^ |
| No | 94 (83.2%) | 78 (82.1%) | 16 (88.9%) |  |
| Yes | 19 (16.8%) | 17 (17.9%) | 2 (11.1%) |  |
|  |  |  |  |  |
| **Formulary/Non-Medical Switch**, N (%) |  |  |  | 1.00^2^ |
| No | 101 (89.4%) | 85 (89.5%) | 16 (88.9%) |  |
| Yes | 12 (10.6%) | 10 (10.5%) | 2 (11.1%) |  |
|  |  |  |  |  |
| **Final Approval**, N (%)  *(Missing = 9)* |  |  |  | 0.35^2^ |
| No | 24 (23.1%) | 22 (25.3%) | 2 (11.8%) |  |
| Yes | 80 (76.9%) | 65 (74.7%) | 15 (88.2%) |  |
|  |  |  |  |  |
| **Delay** (days)  *(Missing = 38)* |  |  |  | 0.02^1^ |
| Mean (SD) | 29.0 (37.4) | 31.7 (39.3) | 15.9 (23.6) |  |
| Median (IQR) | 18 (10, 35) | 21 (10, 40) | 10 (4, 14) |  |
|  |  |  |  |  |
| **Bridge Therapy**, N (%)  *(Missing = 4)* |  |  |  | 0.002^2^ |
| No | 57 (52.3%) | 43 (46.2%) | 14 (87.5%) |  |
| Yes | 52 (47.7%) | 50 (53.8%) | 2 (12.5%) |  |
|  |  |  |  |  |
| **Patient Harm**, N (%)  *(Missing = 4)* |  |  |  | 0.02^2^ |
| No | 44 (40.4%) | 32 (35.2%) | 12 (66.7%) |  |
| Yes | 65 (59.6%) | 59 (64.8%) | 6 (33.3%) |  |
|  |  |  |  |  |
| **Hospitalization**, N (%)  *(Missing = 4)* |  |  |  | 0.01^2^ |
| No | 86 (78.9%) | 68 (74.7%) | 18 (100.0%) |  |
| Yes | 23 (21.1%) | 23 (25.3%) | 0 (0.0%) |  |
|  |  |  |  |  |
| **Administrative time** (minutes)  *(Missing = 37)* |  |  |  | 0.04^1^ |
| Mean (SD) | 288 (385) | 320 (415) | 130 (72) |  |
| Median (IQR) | 180 (100, 300) | 180 (100, 360) | 120 (90, 120) |  |

^1^Wilcoxon rank sum test compared the medians of each quantitative factor between payors.

^2^Fisher exact test compared the level proportions of each categorical factor between payors.

PBM = pharmacy benefit manager

SD = standard deviation, IQR = interquartile range.

**Table S2:** Association of patient characteristics and outcomes with payor barriers.

| **Factor** | Total Patients (N=113) | **Payor Barrier** | | | | | | | | | | | |
| --- | --- | --- | --- | --- | --- | --- | --- | --- | --- | --- | --- | --- | --- |
|  |  | **Prior Authorization** | | | **Dose Restriction** | | | **Step Therapy** | | | **Formulary/Non-Medical Switch** | | |
|  |  | No  (N=21) | Yes  (N=92) | P-value | No  (N=71) | Yes  (N=42) | P-value | No  (N=94) | Yes  (N=19) | P-value | No  (N=101) | Yes  (N=12) | P-value |
| **Age** (years) |  |  |  | 0.90^1^ |  |  | 0.73^1^ |  |  | 0.10^1^ |  |  | 0.72^1^ |
| Mean (SD) | 13.0 (3.5) | 13.3 (3.0) | 13.0 (3.6) |  | 13.0 (3.5) | 13.1 (3.7) |  | 13.3 (3.5) | 11.8 (3.5) |  | 13.1 (3.6) | 12.8 (3.4) |  |
| Median (IQR) | 14 (11, 16) | 14 (11, 16) | 13.5 (11, 16) |  | 13 (11, 16) | 15 (11, 16) |  | 14 (11, 16) | 12 (10, 13) |  | 14 (11, 16) | 13 (11, 16) |  |
|  |  |  |  |  |  |  |  |  |  |  |  |  |  |
| **Sex**, N (%) |  |  |  | 0.47^2^ |  |  | 1.00^2^ |  |  | 0.45^2^ |  |  | 0.37^2^ |
| Female | 59 (52.2%) | 9 (42.9%) | 50 (54.3%) |  | 37 (52.1%) | 22 (52.4%) |  | 51 (54.3%) | 8 (42.1%) |  | 51 (50.5%) | 8 (66.7%) |  |
| Male | 54 (47.8%) | 12 (57.1%) | 42 (45.7%) |  | 34 (47.9%) | 20 (47.6%) |  | 43 (45.7%) | 11 (57.9%) |  | 50 (49.5%) | 4 (33.3%) |  |
|  |  |  |  |  |  |  |  |  |  |  |  |  |  |
| **Medication Requested**, N (%) |  |  |  | 0.51^2^ |  |  | 0.02^2^ |  |  | 0.007^2^ |  |  | 0.03^2^ |
| Infliximab/Inflectra/Avsola | 42 (37.2%) | 8 (38.1%) | 34 (37.0%) |  | 19 (26.8%) | 23 (54.8%) |  | 37 (39.4%) | 5 (26.3%) |  | 34 (33.7%) | 8 (66.7%) |  |
| Adalimumab | 31 (27.4%) | 3 (14.3%) | 28 (30.4%) |  | 24 (33.8%) | 7 (16.7%) |  | 19 (20.2%) | 12 (63.2%) |  | 29 (28.7%) | 2 (16.7%) |  |
| Ustekinemab | 27 (23.9%) | 8 (38.1%) | 19 (20.7%) |  | 17 (23.9%) | 10 (23.8%) |  | 26 (27.7%) | 1 (5.3%) |  | 27 (26.7%) | 0 (0.0%) |  |
| Vedolizumab | 6 (5.3%) | 1 (4.8%) | 5 (5.4%) |  | 4 (5.6%) | 2 (4.8%) |  | 5 (5.3%) | 1 (5.3%) |  | 6 (5.9%) | 0 (0.0%) |  |
| Tofacitinib/Upacitinib | 6 (5.3%) | 1 (4.8%) | 5 (5.4%) |  | 6 (8.5%) | 0 (0.0%) |  | 6 (6.4%) | 0 (0.0%) |  | 4 (4.0%) | 2 (16.7%) |  |
| Risankizumab | 1 (0.9%) | 0 (0.0%) | 1 (1.1%) |  | 1 (1.4%) | 0 (0.0%) |  | 1 (1.1%) | 0 (0.0%) |  | 1 (1.0%) | 0 (0.0%) |  |
|  |  |  |  |  |  |  |  |  |  |  |  |  |  |
| **Insurance vs PBM**, N (%) |  |  |  | 0.02^2^ |  |  | 0.80^2^ |  |  | 0.73^2^ |  |  | 1.00^2^ |
| Insurance | 95 (84.1%) | 21 (100.0%) | 74 (80.4%) |  | 59 (83.1%) | 36 (85.7%) |  | 78 (83.0%) | 17 (89.5%) |  | 85 (84.2%) | 10 (83.3%) |  |
| PBM | 18 (15.9%) | 0 (0.0%) | 18 (19.6%) |  | 12 (16.9%) | 6 (14.3%) |  | 16 (17.0%) | 2 (10.5%) |  | 16 (15.8%) | 2 (16.7%) |  |
|  |  |  |  |  |  |  |  |  |  |  |  |  |  |
| **Alternative Recommendation**, N (%)  *(Missing = 26)* |  |  |  | 0.55^2^ |  |  | 0.28^2^ |  |  | 0.008^2^ |  |  | 0.005^2^ |
| No | 67 (77.0%) | 17 (85.0%) | 50 (74.6%) |  | 44 (73.3%) | 23 (85.2%) |  | 59 (83.1%) | 8 (50.0%) |  | 62 (82.7%) | 5 (41.7%) |  |
| Yes | 20 (23.0%) | 3 (15.0%) | 17 (25.4%) |  | 16 (26.7%) | 4 (14.8%) |  | 12 (16.9%) | 8 (50.0%) |  | 13 (17.3%) | 7 (58.3%) |  |
|  |  |  |  |  |  |  |  |  |  |  |  |  |  |
| **Prior Authorization**, N (%) |  |  |  | – |  |  | 0.003^2^ |  |  | 0.75^2^ |  |  | 0.69^2^ |
| No | 21 (18.6%) | – | – |  | 7 (9.9%) | 14 (33.3%) |  | 17 (18.1%) | 4 (21.1%) |  | 18 (17.8%) | 3 (25.0%) |  |
| Yes | 92 (81.4%) | – | – |  | 64 (90.1%) | 28 (66.7%) |  | 77 (81.9%) | 15 (78.9%) |  | 83 (82.2%) | 9 (75.0%) |  |
|  |  |  |  |  |  |  |  |  |  |  |  |  |  |
| **Dose Restriction**, N (%) |  |  |  | 0.003^2^ |  |  | – |  |  | 0.009^2^ |  |  | 0.53^2^ |
| No | 71 (62.8%) | 7 (33.3%) | 64 (69.6%) |  | – | – |  | 54 (57.4%) | 17 (89.5%) |  | 62 (61.4%) | 9 (75.0%) |  |
| Yes | 42 (37.2%) | 14 (66.7%) | 28 (30.4%) |  | – | – |  | 40 (42.6%) | 2 (10.5%) |  | 39 (38.6%) | 3 (25.0%) |  |
|  |  |  |  |  |  |  |  |  |  |  |  |  |  |
| **Step Therapy**, N (%) |  |  |  | 0.75^2^ |  |  | 0.009^2^ |  |  | – |  |  | 0.69^2^ |
| No | 94 (83.2%) | 17 (81.0%) | 77 (83.7%) |  | 54 (76.1%) | 40 (95.2%) |  | – | – |  | 83 (82.2%) | 11 (91.7%) |  |
| Yes | 19 (16.8%) | 4 (19.0%) | 15 (16.3%) |  | 17 (23.9%) | 2 (4.8%) |  | – | – |  | 18 (17.8%) | 1 (8.3%) |  |
|  |  |  |  |  |  |  |  |  |  |  |  |  |  |
| **Formulary/Non-Medical Switch**, N (%) |  |  |  | 0.69^2^ |  |  | 0.53^2^ |  |  | 0.69^2^ |  |  | – |
| No | 101 (89.4%) | 18 (85.7%) | 83 (90.2%) |  | 62 (87.3%) | 39 (92.9%) |  | 83 (88.3%) | 18 (94.7%) |  | – | – |  |
| Yes | 12 (10.6%) | 3 (14.3%) | 9 (9.8%) |  | 9 (12.7%) | 3 (7.1%) |  | 11 (11.7%) | 1 (5.3%) |  | – | – |  |
|  |  |  |  |  |  |  |  |  |  |  |  |  |  |
| **Final Approval**, N (%)  *(Missing = 9)* |  |  |  | 0.003^2^ |  |  | 0.16^2^ |  |  | 0.76^2^ |  |  | 1.00^2^ |
| No | 24 (23.1%) | 10 (50.0%) | 14 (16.7%) |  | 12 (18.5%) | 12 (30.8%) |  | 21 (24.4%) | 3 (16.7%) |  | 22 (23.7%) | 2 (18.2%) |  |
| Yes | 80 (76.9%) | 10 (50.0%) | 70 (83.3%) |  | 53 (81.5%) | 27 (69.2%) |  | 65 (75.6%) | 15 (83.3%) |  | 71 (76.3%) | 9 (81.8%) |  |
|  |  |  |  |  |  |  |  |  |  |  |  |  |  |
| **Delay** (days)  *(Missing = 38)* |  |  |  | 0.054^1^ |  |  | 0.91^1^ |  |  | 0.49^1^ |  |  | 0.39^1^ |
| Mean (SD) | 29.0 (37.4) | 67.4 (80.9) | 23.0 (20.7) |  | 30.1 (42.0) | 26.7 (27.1) |  | 27.1 (37.8) | 36.3 (36.1) |  | 30.3 (39.1) | 17.9 (14.5) |  |
| Median (IQR) | 18 (10, 35) | 41.5 (14, 90) | 14 (10, 30) |  | 18 (10, 38) | 16.5 (10, 28) |  | 17 (10, 30) | 18 (10, 60) |  | 18 (10, 38) | 17.5 (7, 21) |  |
|  |  |  |  |  |  |  |  |  |  |  |  |  |  |
| **Bridge Therapy**, N (%)  *(Missing = 4)* |  |  |  | 0.47^2^ |  |  | 0.55^2^ |  |  | 0.10^2^ |  |  | 0.55^2^ |
| No | 57 (52.3%) | 9 (42.9%) | 48 (54.5%) |  | 37 (55.2%) | 20 (47.6%) |  | 52 (55.9%) | 5 (31.3%) |  | 52 (53.6%) | 5 (41.7%) |  |
| Yes | 52 (47.7%) | 12 (57.1%) | 40 (45.5%) |  | 30 (44.8%) | 22 (52.4%) |  | 41 (44.1%) | 11 (68.8%) |  | 45 (46.4%) | 7 (58.3%) |  |
|  |  |  |  |  |  |  |  |  |  |  |  |  |  |
| **Patient Harm**, N (%)  *(Missing = 4)* |  |  |  | 0.80^2^ |  |  | 0.23^2^ |  |  | 0.30^2^ |  |  | 0.76^2^ |
| No | 44 (40.4%) | 7 (36.8%) | 37 (41.1%) |  | 31 (44.9%) | 13 (32.5%) |  | 34 (37.8%) | 10 (52.6%) |  | 40 (41.2%) | 4 (33.3%) |  |
| Yes | 65 (59.6%) | 12 (63.2%) | 53 (58.9%) |  | 38 (55.1%) | 27 (67.5%) |  | 56 (62.2%) | 9 (47.4%) |  | 57 (58.8%) | 8 (66.7%) |  |
|  |  |  |  |  |  |  |  |  |  |  |  |  |  |
| **Hospitalization**, N (%)  *(Missing = 4)* |  |  |  | 1.00^2^ |  |  | 0.81^2^ |  |  | 0.35^2^ |  |  | 0.71^2^ |
| No | 86 (78.9%) | 15 (78.9%) | 71 (78.9%) |  | 55 (79.7%) | 31 (77.5%) |  | 69 (76.7%) | 17 (89.5%) |  | 77 (79.4%) | 9 (75.0%) |  |
| Yes | 23 (21.1%) | 4 (21.1%) | 19 (21.1%) |  | 14 (20.3%) | 9 (22.5%) |  | 21 (23.3%) | 2 (10.5%) |  | 20 (20.6%) | 3 (25.0%) |  |
|  |  |  |  |  |  |  |  |  |  |  |  |  |  |
| **Administrative time** (minutes)  *(Missing = 37)* |  |  |  | 0.12^1^ |  |  | 0.86^1^ |  |  | 0.66^1^ |  |  | 0.66^1^ |
| Mean (SD) | 288 (385) | 577 (768) | 244 (273) |  | 257 (294) | 345 (513) |  | 307 (419) | 201 (155) |  | 278 (362) | 359 (548) |  |
| Median (IQR) | 180 (100, 300) | 240 (120, 600) | 155 (90, 300) |  | 180 (100, 300) | 180 (90, 360) |  | 180 (100, 300) | 120 (100, 240) |  | 180 (90, 300) | 120 (120, 300) |  |

^1^Wilcoxon rank sum test compared the medians of each quantitative factor between levels of each payor barrier.

^2^Fisher exact test compared the level proportions of each categorical factor between levels of each payor barrier.

PBM = pharmacy benefit manager

SD = standard deviation, IQR = interquartile range.


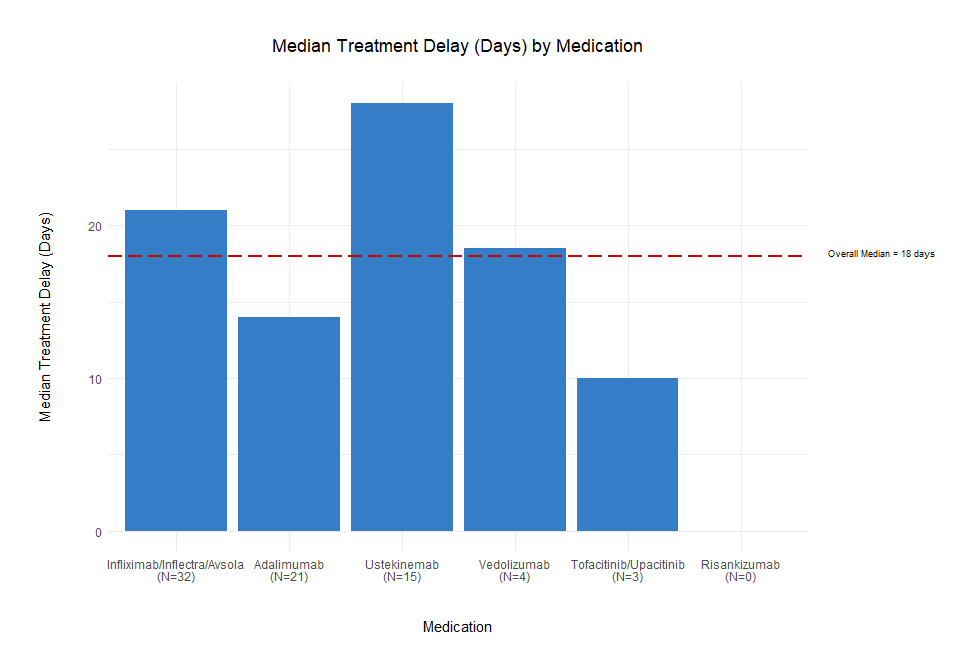


**Figure S3.** Median delay time (days) to treatment with biologics medications for pediatric patients with inflammatory bowel disease (IBD) encountering payor barriers.


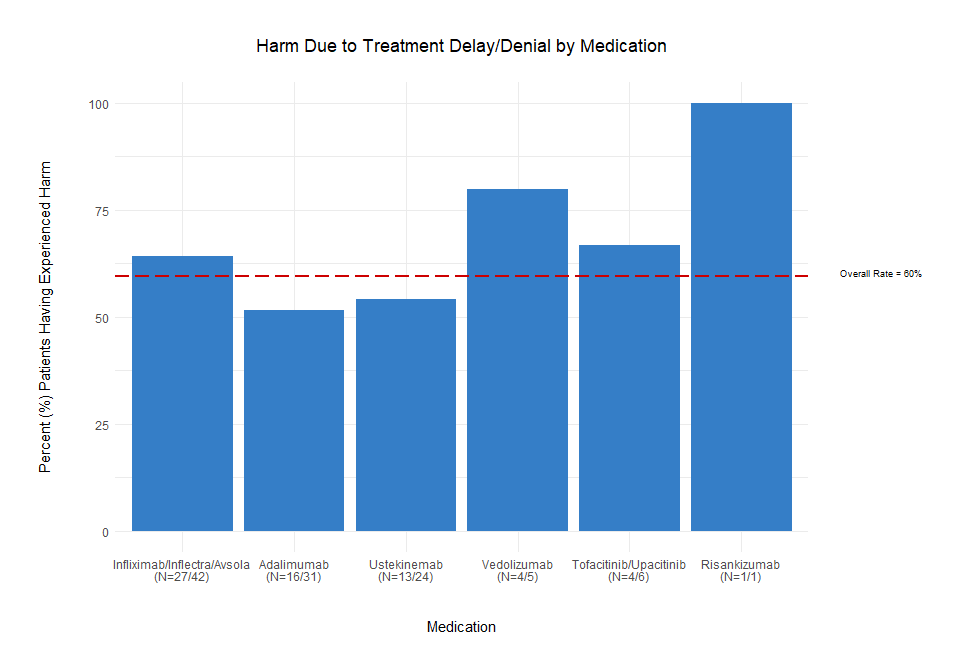


**Figure S4:** Pediatric patients with inflammatory bowel disease (IBD) experiencing harm due to payor delay/denial of treatment with biologics medications.


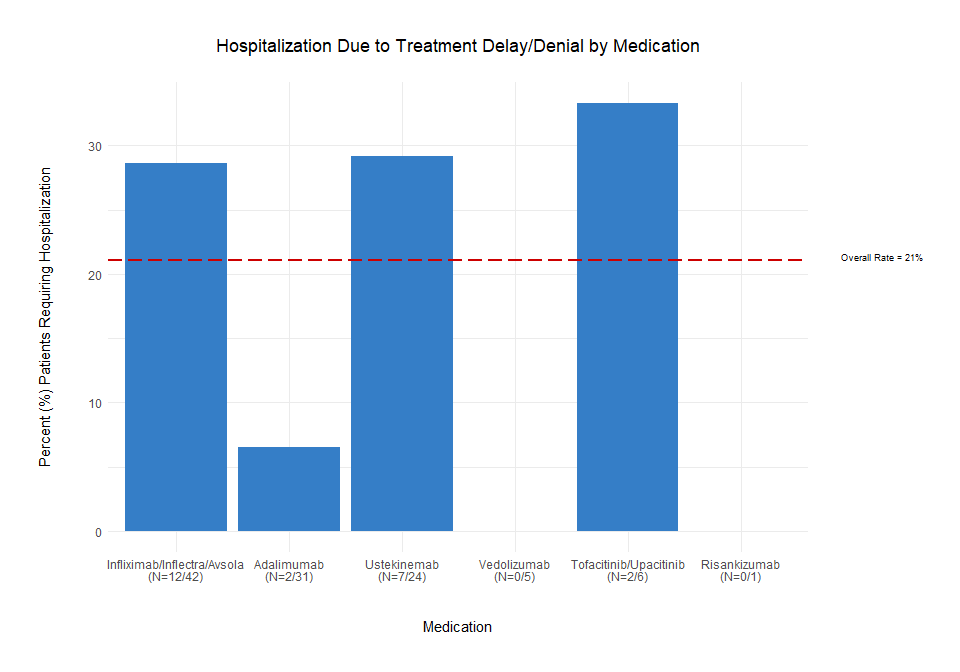


**Figure S5:** Pediatric patients with inflammatory bowel disease (IBD) hospitalized due to payor delay/denial of treatment with biologics medications.


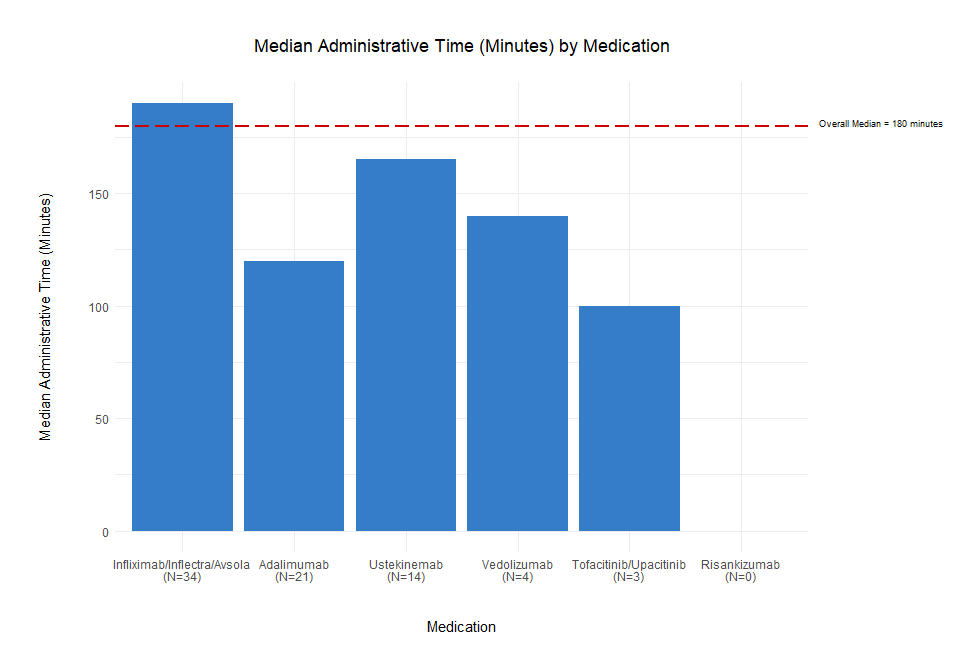
 **Figure S6:** Median administrative time (minutes) required to respond to payor barriers to treatment of pediatric patients with inflammatory bowel disease (IBD) with biologics medications.

**Survey Questions** Survey provided to the pediatric gastroenterology provider Listserv.

| **1)** | **Patient Age** |  |
| --- | --- | --- |
| **2)** | **Patient Gender** | Female   Male   Undifferentiated  [reset](javascript:;) |
| **3)** | **Patient Location (State)** |  |
| **4)** | **Provider Location (State)** |  |
| **5)** | **Denial was made by** | Insurance Provider   Physician Benefit Manager (PBM)  [reset](javascript:;) |
| **6)** | **Name of insurance company or PBM** |  |
| **7)** | **Medication Prescribed** |  |
| **8)** | **Alternative Medication Recommended (if applicable)** |  |
| **9)** | **Reason for Denial (brief description- please add if infusion site denied as well)** |  [Expand](javascript:;) |
| **10)** | **Medication Utilization Barrier** | Prior Authorization   Step Therapy   Formulary Restriction   Dose Restriction (dose or interval)   Non-Medical Switch   Other  |
| **11)** | **Delay in Starting Medication (Days)** |  |
| **12)** | **Additional Bridge Therapy Required (Steroids, Antibiotics, Nutrition, etc.)** | Yes   No  [reset](javascript:;) |
| **13)** | **Was therapy ultimately approved?** | Yes   No  [reset](javascript:;) |
| **14)** | **Estimated total administrative time spent working on approval (total minutes)** |  |
| **15)** | **Please describe if this delay/denial harmed your patient (quality of life, hospitalization, surgery, etc/)** |  |
